# Sexual cannibalism and population viability

**DOI:** 10.1002/ece3.4155

**Published:** 2018-06-24

**Authors:** Adam M. Fisher, Stephen J. Cornell, Gregory I. Holwell, Tom A. R. Price

**Affiliations:** ^1^ Institute of Integrative Biology University of Liverpool Liverpool UK; ^2^ Department of Biology University of Auckland Auckland New Zealand

**Keywords:** arachnid, cannibalism, extinction, extinction vortex, mantis, population growth, population survival

## Abstract

Some behaviours that typically increase fitness at the individual level may reduce population persistence, particularly in the face of environmental changes. Sexual cannibalism is an extreme mating behaviour which typically involves a male being devoured by the female immediately before, during or after copulation, and is widespread amongst predatory invertebrates. Although the individual‐level effects of sexual cannibalism are reasonably well understood, very little is known about the population‐level effects. We constructed both a mathematical model and an individual‐based model to predict how sexual cannibalism might affect population growth rate and extinction risk. We found that in the absence of any cannibalism‐derived fecundity benefit, sexual cannibalism is always detrimental to population growth rate and leads to a higher population extinction risk. Increasing the fecundity benefits of sexual cannibalism leads to a consistently higher population growth rate and likely a lower extinction risk. However, even if cannibalism‐derived fecundity benefits are large, very high rates of sexual cannibalism (>70%) can still drive the population to negative growth and potential extinction. Pre‐copulatory cannibalism was particularly damaging for population growth rates and was the main predictor of growth declining below the replacement rate. Surprisingly, post‐copulatory cannibalism had a largely positive effect on population growth rate when fecundity benefits were present. This study is the first to formally estimate the population‐level effects of sexual cannibalism. We highlight the detrimental effect sexual cannibalism may have on population viability if (1) cannibalism rates become high, and/or (2) cannibalism‐derived fecundity benefits become low. Decreased food availability could plausibly both increase the frequency of cannibalism, and reduce the fecundity benefit of cannibalism, suggesting that sexual cannibalism may increase the risk of population collapse in the face of environmental change.

## 1 INTRODUCTION

Sexual cannibalism typically involves a female devouring a conspecific male immediately before, during or immediately after copulation. Although sexual cannibalism has been observed in a wide variety of predatory invertebrates, the majority of evidence comes from studies involving spiders or mantids (Elgar, 1992). To date, research into sexual cannibalism has largely focussed on discovering its adaptive function. Currently, there are three main hypotheses that suggest why sexual cannibalism is maintained in nature: (1) adaptive foraging—females devour males in order to gain essential nutrients and are more likely to do so when starved (Barry, Holwell, & Herberstein, 2008; Hurd et al., 1994; Roggenbuck, Pekár, & Schneider, 2011); (2) mate choice—sexual cannibalism represents an extreme form of mate choice in which non‐preferred males are devoured to prevent copulation (Elgar, Schneider, & Herberstein, 2000; Hebets, 2003; Persons & Uetz, 2005; for a review see: Prenter, MacNeil, & Elwood, 2006); (3) aggressive spillover—adult female aggression towards conspecific males is a by‐product of strong selection for juvenile aggression (Arnqvist, 1992; Henriksson, 1997; Johnson & Sih, 2005).

The extent to which females cannibalise males can vary greatly between individuals, species and taxonomic groups. For example, female *Dolomedes triton* (six‐spotted fishing spider) show individually consistent differences in how likely they are to cannibalise males (Johnson & Sih, 2005). Female diet can create large variation in sexual cannibalism rates within species; female *Pseudomantis albofimbriata* (false garden mantis) cannibalise males at an average of 89% of encounters when starved, compared to 0% of encounters when satiated (Barry et al., 2008). Female attack rates in *Miomantis caffra* (springbok mantis) are not significantly affected by diet, with females cannibalising males at an average rate of 61% (Walker & Holwell, 2015). A field study on *Mantis religiosa* (the European mantis) indicates that females cannibalise males in only 31% of encounters (Lawrence, 1992). As well as variation in the overall rate of sexual cannibalism, variation in the probability of cannibalism occurring before or after copulation also differs between species. For example, female free‐living hunting spiders are known for their high tendency to attack males prior to copulation (see: Persons & Uetz, 2005 [wolf spiders] and Arnqvist, 1992; Johnson & Sih, 2005 [fishing spiders]). Conversely, females of some web‐dwelling spiders typically wait until after copulation before attempting to devour the male (Forster, 1992; Herberstein, Schneider, & Elgar, 2002; Schneider & Lesmono, 2009).

Although the costs and benefits of sexual cannibalism for the individuals of a species have been thoroughly investigated (Barry et al., 2008; Brown & Barry, 2016; Newman & Elgar, 1991; Schneider & Elgar, 2001; Schwartz, Wagner, & Hebets, 2016), there are very few studies that attempt to address the population‐level consequences of sexual cannibalism. There are two main negative effects that sexual cannibalism may have on population ecology. First, in all instances of sexual cannibalism, an adult male is removed from the population, which over time will deplete the availability of reproductive males in the population (Hurd et al., 1994; Lawrence, 1992). Second, in the case of pre‐copulatory cannibalism, an adult male is removed from the population and both the male and the female miss out on copulation and the fertilisation of the female's eggs. Both of these effects of sexual cannibalism are likely to increase the proportion of adults in the population, both male and female, that die as virgins. As a result of high virgin death rates, the population may be more vulnerable to ecological decline via mechanisms such as reduced population growth rate (*r*) (Anthony & Blumstein, 2000; Miller et al., 2009).

Understanding the effects of individual behaviour on species ecology is important for determining population viability (Anthony & Blumstein, 2000; Blumstein, 1998). Here, we provide a theoretical framework that examines the potential effect of sexual cannibalism on population growth rate and population extinction risk. We also compare the population‐level effects of pre‐ and post‐copulatory cannibalism separately to give a more holistic overview of how sexual cannibalism can impact on species ecology.

## 2 METHODS

For robustness, we used two separate modelling approaches to address our central question of how sexual cannibalism affects population viability. We first programmed a discrete‐time mathematical model. Mathematical models are noted for their conciseness and ability to generalise theoretical findings for a multitude of biological systems; however for tractability they often ignore stochasticity that can be generated by natural variation. As such, we also constructed an individual‐based model (IBM). IBMs consider individuals as discrete entities and incorporate uncertainty into a model. Due to the fact that system‐specific behaviours are explicitly defined in IBMs, they are often seen as being more realistic relative to other types of theoretical model (Grimm, 1999). Both models were run in R version 3.4.3 (R Core Team, 2017). Code for both models is available at datadryad (doi:10.5061/dryad.gr5hc09).

### 2.1 Mathematical model

Our mathematical model is spatially implicit and assumes no migration of individuals. We assume males (*M*) search for mates continually until they are eaten by a female or reach their maximum mating quota (*h*). Males are always successful at finding a mate; we justify this assumption in sexually cannibalistic systems using the fact that, due to sexual cannibalism, adult death rates are likely to be greater for males than for females. As such, this will reduce the probability of sexually receptive females becoming scarce in relation to males; thus a significant reduction in male mate‐finding rate is unlikely to occur. We assume an initial 1:1 adult sex ratio, based on Fisher's principle, and supported by field data from two sexually cannibalistic species (Arnqvist & Henriksson, 1997; Lawrence, 1992). Because our model considers a closed population (migration rate = 0) and assumes an even adult sex ratio, we can regard the average overall mating rate for males and females as equal in a generation (i.e., if the average number of matings per male is two, then the average number of matings per female must also be two).

We gave *h* a constant value of 2, this value is in concordance with evidence from empirical studies, which suggest that the maximum number of lifetime matings for males in cannibalistic species is often two (Fromhage & Schneider, 2006; Herberstein, Gaskett, Schneider, Vella, & Elgar, 2005). In addition, we provide figures displaying how our model output is affected by varying the value of *h* in the Supporting Information. The probability of both pre‐copulatory cannibalism (*c*_1_) and post‐copulatory cannibalism (*c*_2_) was given separate values so that the ecological significance of both pre‐ and post‐copulatory cannibalism could be investigated independently. After locating a female, there was probability *k* that the male would have the opportunity to mate. For the figures presented in the main manuscript *k* is kept at a constant value of 1, however figures displaying how our results are affected by varying *k* are provided in the Supporting Information. The total number of matings that have taken place over the breeding season, *m*, is therefore given by

(1) $$m=kM\left(1-c_{1}\right)\left(1+\sum_{j=1}^{h-1}{\left\{\left(1-c_{1}\right)\left(1-c_{2}\right)\right\}}^{j}\right).$$

Since the female population is not explicitly defined in the mathematical model, fecundity was quantified in terms of the number of males produced per mating over the course of a breeding season. The minimum number of males produced per copulation was defined as *a*. Fecundity benefits (*b*) could be gained from both pre‐copulatory cannibalism and post‐copulatory cannibalism. The cannibalism derived fecundity benefits were assumed to increase linearly with the probability of sexual cannibalism occurring. As there is no conclusive evidence to state that either pre‐ or post‐copulatory cannibalism has a greater potential to increase female fecundity, we assume that both cannibalism rates influence fecundity equally. As such, the mean number of males produced per copulation is

(2) $$f=(a+bac_{1}+\;bac_{2}).$$

Therefore, the mean *N*_*t* + 1_ is

(3) $$N_{t+1}=mf=kM\left(1-c_{1}\right)\left(1+\sum_{j=1}^{h-1}{\left\{\left(1-c_{1}\right)\left(1-c_{2}\right)\right\}}^{j}\right)\left(a+bac_{1}+bac_{2}\right).$$

### 2.2 Individual‐based model

A stochastic individual‐base model, running on a daily cycle, was also used to simulate the lifecycle of a sexually cannibalistic system. Each day, males and females had a constant probability γ of dying via a means other than sexual cannibalism. Individual males were then given the opportunity to find a mate. The probability *L*_*m*_ of finding a mate was assumed to have a hyperbolic relationship with the number of receptive females present in the population. There is evidence for hyperbolic resource‐finding relationships in other arthropod species (Hassell, Lawton, & Beddington, 1976). As such, male mate location probability was defined as

(4) $$Lm=\frac{h\left(\frac{R_{t}}{R_{t}+\theta }\right)}{\delta },$$

where δ denotes total breeding season length (days), *R*_*t*_ is the number of receptive females at time *t*,* h* is the maximum number of matings a male can achieve in his lifetime and θ is a constant used to determine the gradient of the mate‐finding curve. Females were assumed to become non‐receptive once mated; however after a gestation period (μ) they became receptive again so that when *t *> μ,

(5) $$R_{t}=F_{t}-\;m_{\mathrm{tot}(t)}+m_{t-\mu },$$

where *F*_*t*_ is the total number of females at time *t*,* m*_tot(*t*)_ is the total number of matings that had taken place up until time *t*, and *m*_*t *− μ_ is the number of matings that took place at time *t *− μ. After locating a mate, males can be cannibalised before copulation with probability *L*_*m*1_ = *L*_*m*_ *c*_1_, copulate successfully with probability *L*_*m*2_ = *L*_*m*_(1 − *c*_1_), copulate and then be cannibalised with probability *L*_*m*3_ = *L*_*m*2_ *c*_2_, or copulate and then survive with probability *L*_*m*4_ = *L*_*m*2_(1 − *c*_2_). The probabilities of the aforementioned events occurring were then used to create a multinomial random number vector containing the number of males that were assigned to each event. The simulation ran for a series of δ days, and the number of successful mating events that occurred throughout the season was summed to give *m*. Average female fecundity (*f*) was defined in the same way as for the mathematical model (Equation (2)). Values for *f* were varied following a Poisson distribution to imitate natural variation in female fecundity. The number of adults present at *N*_*t + *1_ is then calculated by *mf*. Population growth rate is then calculated by *N*_*t*+1_/*N*_*t*_. To minimise any influence that initial population size may have on population growth rate, the above process was repeated for a range of initial population sizes to determine a reliable mean value for population growth rate.

## 3 RESULTS

Please note: the absolute values for population growth rate produced by these models are sensitive to initial parameter values. Because initial parameter settings cannot be made appropriate for all of the species that the model is relevant to, any conclusions drawn from absolute values would be unreliable. As such, the *y*‐axis in this section is often not fully labelled. Code for the models is available at datadryad.org for production of results for specific initial parameter values.

**Figure 1:**
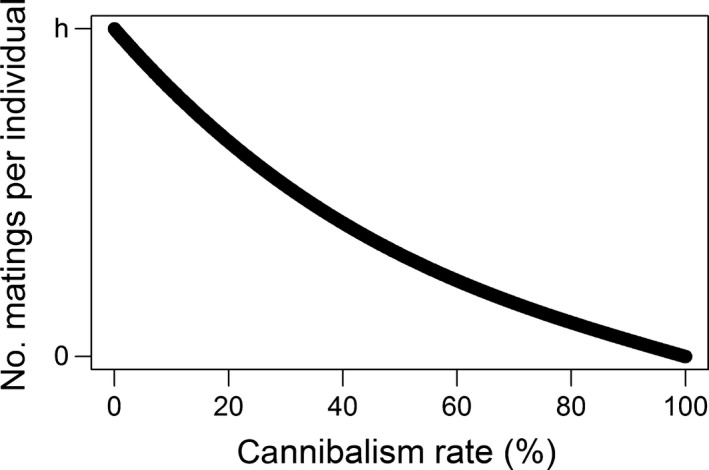
The average number of matings per individual declines in response to increasing sexual cannibalism rate. Here, it is assumed that pre‐ and post‐copulatory cannibalism rates are equal. *h* is the average maximum number of lifetime matings per individual

Sexual cannibalism causes adult male deaths and, in some cases, leads to both the male and female missing out on copulation. Our models show that an overall increase in sexual cannibalism leads to consistently lower mating rates (Figure 1). This was true for both the mathematical model and the individual‐based model (IBM). Although this result is unsurprising, it is a necessary premise to support our further findings.

### 3.1 Sexual cannibalism and population growth rate

**Figure 2:**
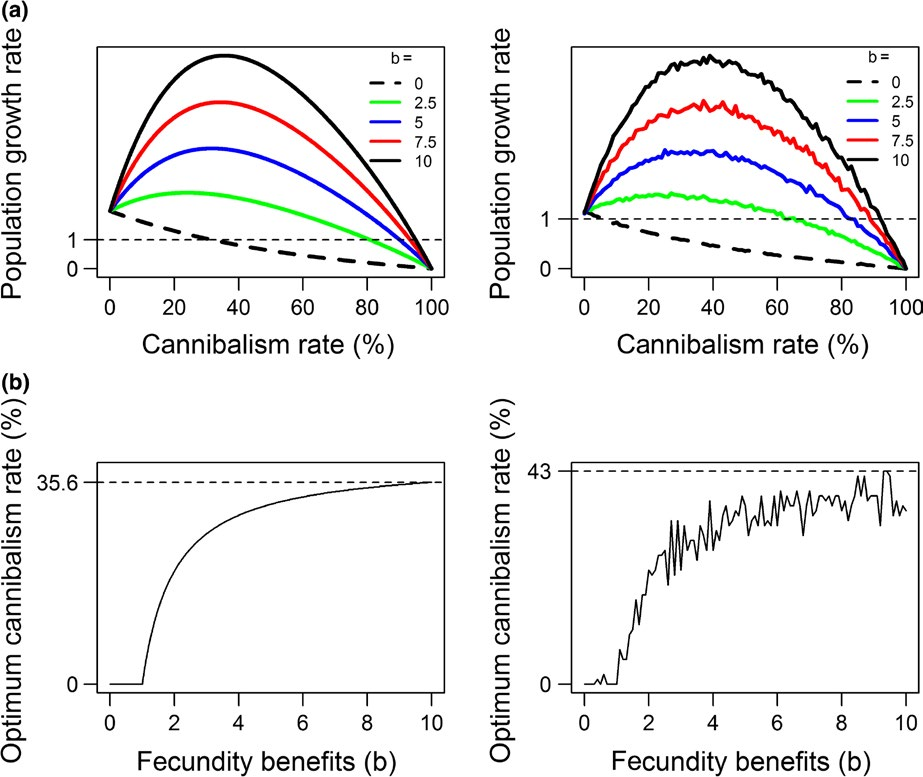
(a) Variation in population growth rate in response to cannibalism rate and the fecundity benefits of sexual cannibalism (*b*). The horizontal dashed line represents the extinction threshold. (b) Variation in optimum cannibalism rate for population growth in response to increased *b* value. Pre‐ and post‐copulatory cannibalism rates are assumed to be equal for these illustrative results. Results from the mathematical model are displayed on the left, IBM results on the right

Our mathematical and individual‐based model showed very similar trends for the effect of sexual cannibalism on population growth rate (Figure 2), both in the presence and in the absence of cannibalism‐derived fecundity benefits (*b*). In the absence of any type of fecundity benefit (*b* = 0), sexual cannibalism consistently decreases population growth rate. However, in the presence of fecundity benefits, sexual cannibalism can increase population growth rate, with increased fecundity benefits causing higher growth rates across all rates of sexual cannibalism (Figure 2a). Increasing the value of *b* caused a type II functional response in the rate of sexual cannibalism that gave the highest growth rate value (Figure 2b), that is, the rate of increase in the optimal cannibalism rate for population growth decreases as *b* becomes larger. The highest optimum cannibalism rate was 36% for the mathematical model and 43% for the IBM.

Higher rates of sexual cannibalism caused population growth rate to decline and eventually fall below the extinction threshold, below which the population is expected to decline in size each generation (Figure 2a). Lower *b* values caused the curves to fall below the extinction threshold sooner than curves representing higher *b* values. For example, when *b* = 0, the mathematical model predicted that a population would go extinct when the rate of sexual cannibalism is >32%. The individual‐based model predicted a threshold of >6% when *b* = 0. However, when a positive *b* value was used (*b *≥ 2.5), populations would not go extinct until cannibalism rates became >82% in the mathematical model and >66% in the IBM.

### 3.2 Pre‐ versus post‐copulatory cannibalism

**Figure 3:**
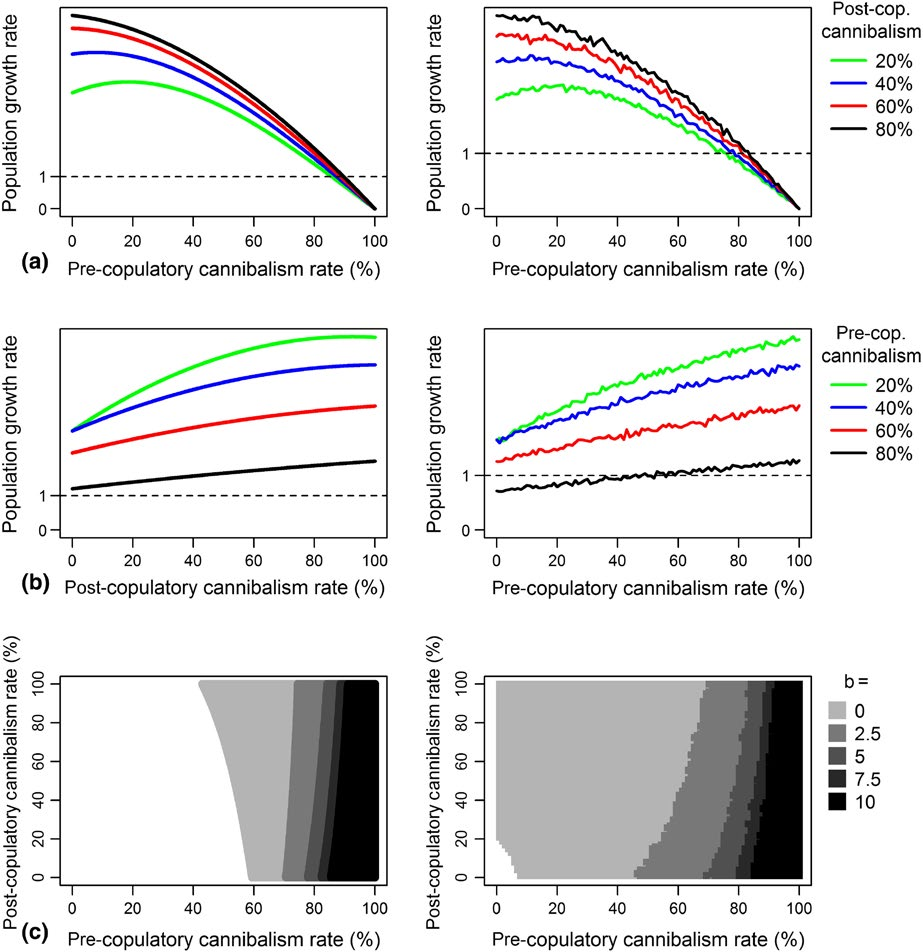
(a, b) Variation in population growth rate in response to changes in both pre‐ and post‐copulatory cannibalism rate (*b* = 5). In Figure 3a, pre‐copulatory cannibalism rate is on a continuous scale while each individual line represents a fixed value for post‐copulatory cannibalism rate. For Figure 3b, post‐copulatory cannibalism rate is varied whilst pre‐copulatory cannibalism rate is fixed. (c) Effect of pre‐ and post‐copulatory cannibalism rate and the amount of cannibalism derived benefits (*b*) on population extinction zone (shaded areas). Results from the mathematical model are displayed on the left, IBM results on the right

When observed separately, pre‐ and post‐copulatory had different effects on population growth rate (Figure 3a,b). Results from both the mathematical model and the IBM showed that pre‐copulatory cannibalism had a largely negative effect on population growth rate (Figure 3a). The mathematical model and the IBM showed a consistent decrease in population growth rate in response to an increase in pre‐copulatory cannibalism rate. Both models show that changes in post‐copulatory cannibalism rate have only a small effect on population growth rate, and that this effect decreases as pre‐copulatory cannibalism rate increases (i.e., the separate lines merge, Figure 3a). Both the mathematical model and the IBM show that post‐copulatory sexual cannibalism consistently increases population growth rate (Figure 3b). In both models, higher pre‐copulatory cannibalism rates led to consistently lower growth rates (Figure 3b).

Whether the population was able to remain above the extinction threshold was largely determined by the rate of pre‐copulatory sexual cannibalism, with post‐copulatory cannibalism having only a small effect (Figure 3c). This was true for both the mathematical and IBM. In the presence of a positive *b* value (*b* ≥ 2.5), extinction only occurred at intermediate to high rates of pre‐copulatory sexual cannibalism. A pre‐copulatory cannibalism rate of >75% would cause a population to go extinct in the mathematical model, in the IBM a pre‐copulatory cannibalism rate of >≈ 48% would cause population extinction. Removing the fecundity benefits of sexual cannibalism (*b* = 0) sees the extinction threshold shift so that lower rates of pre‐copulatory cannibalism can cause extinction, even when post‐copulatory cannibalism rates are low. This was particularly true of the IBM in which only very low values of pre‐ and post‐copulatory cannibalism did not cause population extinction. A *b* value of zero also saw the effect of post‐copulatory cannibalism on population growth rate change from positive to negative (Figure 3c).

## 4 DISCUSSION

Using theoretical models, we have demonstrated that sexual cannibalism can have both positive and negative impacts on population growth rate. Intermediate rates of sexual cannibalism give the highest population growth rate (*r*) values when cannibalism‐derived fecundity benefits (*b*) are present. This finding is likely to represent a behavioural trade‐off in which nutrients gained from intermediate cannibalism rates lead to a higher rate of offspring production, despite cannibalism reducing the number of matings taking place in the population. However, we have also shown that high rates of sexual cannibalism can reduce population growth below the replacement rate, potentially leading to population extinction, particularly when the fecundity benefit of cannibalism is low. Pre‐copulatory cannibalism was far more likely to reduce population growth rate than post‐copulatory cannibalism. Our IBM generated lower absolute values for population growth rate than the mathematical model, this was likely due to female supply being limited in the IBM but not in the mathematical model. However, the overall trends produced were very similar for both models.

### 4.1 When does sexual cannibalism increase the productivity of a population?

Unsurprisingly, if cannibalism does not improve female fecundity, cannibalism always lowers population growth (Figure 2). Thus, fecundity benefits make it more likely for sexually cannibalistic mating systems to persist in nature. There is substantial evidence that sexual cannibalism increases lifetime fecundity for the female, and sometimes the male (Andrade, 2003; Brown & Barry, 2016). Often this is thought to occur through increased egg production owing to extra nutrients derived from the cannibalised male (Brown & Barry, 2016; Spence, Zimmermann, & Wojcicki, 1996). For example, female *Pseudomantis albofimbriata* that cannibalise males are known to increase their ootheca mass by up to 40% when compared to females that do not cannibalise (Barry et al., 2008). More recently, it was shown that female dark fishing spiders (*Dolomedes tenebrosus*) that cannibalised males produced twice as many offspring as females that did not cannibalise or were given an alternative post‐copulatory meal (Schwartz et al., 2016).

Another way in which sexual cannibalism may increase individual fecundity is via improved offspring quality (Berning et al., 2012; Pruitt et al., 2014). There is evidence that in some species sexual cannibalism is used as a method of mate choice (for a review see: Prenter et al., 2006); as such, cannibalistic females may be able to mate with higher quality males and produce offspring that are more viable. In both *Schizocosa ocreata* (wolf spiders) and *Araneus diadematus* (garden‐orb spiders), females are more likely to exhibit pre‐copulatory cannibalism on males that are relatively small (Persons & Uetz, 2005). Similarly, in the fishing spider *Dolomedes fimbriatus*, larger males are more likely to evade pre‐copulatory cannibalism attempts by females and are therefore more likely to copulate (Arnqvist, 1992; Kralj‐Fišer et al., 2016). Sexual cannibalism may also improve offspring viability indirectly through aggressive spillover, in which sexually cannibalistic behaviour is linked to greater feeding voracity as a juvenile, which in turn leads to higher juvenile survival (Arnqvist, 1992; Henriksson, 1997; Johnson & Sih, 2005). As such, sexually cannibalistic adults may produce more voracious offspring that survive better. Both increased egg production and improved offspring quality represent mechanisms by which sexual cannibalism can be beneficial for individuals.

Our results suggest that intermediate rates of sexual cannibalism yield the highest population growth rates when pre‐ and post‐copulatory cannibalism rates are equal (Figure 2a: 24%–26% in the mathematical model, 18%–52% in the IBM). Evidence from field and laboratory studies suggest that cannibalism rates often fall into this intermediate range. In the wild, European mantis (*Mantis religiosa*) females cannibalised potential mates in 31% of encounters (Lawrence, 1992), with similar rates seen in other mantids (*Hierodula membranacea*, Birkhead, Lee, & Young, 1988; *T. sinensis*, Lelito & Brown, 2006). Evidence from studies involving spiders also demonstrates a tendency for females to cannibalise males at an intermediate rate (*Nephila plumipes*, 56%–61% in Schneider & Elgar, 2001; *D. triton*, 21%–53% in Johnson, 2001). It may be that intermediate levels of sexual cannibalism represent an optimal trade‐off between reduced mating rate and increased reproductive output per mating, and are thus selected for at the individual level. Alternatively, it could be that species or populations in which high levels of sexual cannibalism are selected for at the individual level are indeed at a higher risk of extinction and so do not persist.

Our results show that even high rates of post‐copulatory cannibalism are not detrimental to population growth when cannibalism provides fecundity benefits (Figure 3b). Post‐copulatory cannibalism may represent the “best of both worlds” for females in terms of reproductive output, as it allows the female to secure a mate and potentially increase egg production through devouring the male. In some species, strict post‐copulatory cannibalism is apparent, with females devouring the male before copulation only in rare cases. For example, female orb‐web spiders of the genus *Argiope* typically only attack the male after he has successfully mounted (Schneider & Lesmono, 2009). Although post‐copulatory cannibalism has the potential to reduce the lifetime number of matings for males, males of some species are complicit in being devoured by the female after copulation has taken place, including the redback spider (*Latrodectus hassellti*) (Andrade, 2003), the dark fishing spider (*Dolomedes tenebrosus*) (Schwartz et al., 2016) and the wasp spider (*Argiope bruennichi*) (Fromhage, Uhl, & Schneider, 2003). It is theorised that the increase in paternal output the male receives by allowing himself to be eaten by the female offsets the cost of reducing his lifetime number of matings (Buskirk, Frohlich, & Ross, 1984).

### 4.2 When is sexual cannibalism likely to be detrimental to population survival?

Our models indicate that scenarios in which pre‐ and post‐copulatory sexual cannibalism rates are high can lead to low population growth (Figure 2a) and potentially lead to population extinction (Figures 2a and 3c), even if the fecundity benefits of cannibalism are high. As such, it would seem unlikely that populations in which individuals demonstrate such high cannibalism rates would persist in nature. However, environmental conditions can substantially increase cannibalism rates. Laboratory studies have found tenfold increases in cannibalism rates when females are starved (*Hierodula membranacea*: Birkhead et al., 1988; *Stagmomantis limbata:* Maxwell, Gallego, & Barry, 2010), suggesting that females in poor‐quality habitats, where nutrients are scarce, would cannibalise more frequently. Rates of sexual cannibalism may also increase if there is a decrease in male quality in systems where sexual cannibalism acts as mate selection in which females cannibalise unattractive males (Arnqvist, 1992; Persons & Uetz, 2005). For example, in the wolf spider *Hogna helluo* rates of sexual cannibalism rate can increase from 0% to 80% as male size relative to the female decreases (Wilder & Rypstra, 2008). Therefore if males that develop in poor‐quality habitats do not grow to an adequate size, sexual cannibalism rates may become elevated.

Sexual cannibalism is more likely to reduce population growth below the replacement rate when fecundity benefits are low (Figures 2a and 3c). Hence, the ecological viability of a cannibalistic population may be highly dependent on the nutritional value of males. It is possible that males that develop in poor‐quality habitats are less nutritious and therefore provide less of a fecundity benefit for females. As of yet there are no studies that test this theory. Further experimental investigation into how environmental factors affect male nutritional value quality is needed in order to establish a link between habitat quality and the population‐level effects of sexual cannibalism.

Pre‐copulatory cannibalism was shown to be largely detrimental for population growth rate (Figure 3a). An increase in a female's tendency to cannibalise males before copulation may therefore be detrimental for population growth rate. Although there is little evidence on whether local conditions can increase pre‐copulatory cannibalism in nature, a recent paper has shown that pre‐copulatory cannibalism rates in female wolf spiders increased significantly in response to feeding regime and relative male size in the laboratory (Gavín‐Centol, Kralj‐Fišer, De Mas, Ruiz‐Lupión, & Moya‐Laraño, 2017). Alternatively, if sexual cannibalism occurs due to spillover of juvenile aggression, then poor habitats may select for highly aggressive juveniles (Johnson & Sih, 2005), thereby increasing the rate of adult cannibalism. Thus, if reducing habitat quality does in fact increase female hunger and reduce male size, then not only may we see an increase in cannibalism rate overall, but specifically an increase in pre‐copulatory cannibalism, with potentially greater impacts on population viability.

In summary, our models suggest that populations may suffer increased extinction risk when (1) cannibalism rates are high, (2) fecundity benefits of cannibalism are low, and (3) there is a shift from post‐copulatory to pre‐copulatory cannibalism. There are reasons to believe all three processes are likely to occur when habitat quality is declining. This may make sexually cannibalistic species particularly vulnerable to environmental change.

## CONFLICT OF INTEREST

None declared.

## AUTHOR CONTRIBUTIONS

All authors contributed to the idea for the project, the design and interpretation of the models, and wrote the MS. AMF conceived the project idea, all authors contributed to the design and interpretation of the models, the models were coded by AMF and SJC, all authors contributed to the writing of the manuscript.

## References

[ece34155-bib-0001] Andrade, M. C. (2003). Risky mate search and male self‐sacrifice in redback spiders. Behavioral Ecology, 14(4), 531–538. 10.1093/beheco/arg015

[ece34155-bib-0002] Anthony, L. L. , & Blumstein, D. T. (2000). Integrating behaviour into wildlife conservation: The multiple ways that behaviour can reduce N_e_ . Biological Conservation, 95(3), 303–315. 10.1016/S0006-3207(00)00037-9

[ece34155-bib-0003] Arnqvist, G. (1992). Courtship behavior and sexual cannibalism in the semi‐aquatic fishing spider, *Dolomedes fimbriatus* (Clerck) (Araneae: Pisauridae). Journal of Arachnology, 20(3), 222–226.

[ece34155-bib-0004] Arnqvist, G. , & Henriksson, S. (1997). Sexual cannibalism in the fishing spider and a model for the evolution of sexual cannibalism based on genetic constraints. Evolutionary Ecology, 11(3), 255–273. 10.1023/A:1018412302621

[ece34155-bib-0005] Barry, K. L. , Holwell, G. I. , & Herberstein, M. E. (2008). Female praying mantids use sexual cannibalism as a foraging strategy to increase fecundity. Behavioral Ecology, 19(4), 710–715. 10.1093/beheco/arm156

[ece34155-bib-0006] Berning, A. W. , Gadd, R. D. , Sweeney, K. , MacDonald, L. , Eng, R. Y. , Hess, Z. L. , & Pruitt, J. N. (2012). Sexual cannibalism is associated with female behavioural type, hunger state and increased hatching success. Animal Behaviour, 84(3), 715–721. 10.1016/j.anbehav.2012.06.030

[ece34155-bib-0007] Birkhead, T. R. , Lee, K. E. , & Young, P. (1988). Sexual cannibalism in the praying mantis *Hierodula membranacea* . Behaviour, 106(1), 112–118. 10.1163/156853988X00115

[ece34155-bib-0008] Blumstein, D. T. (1998). Female preferences and effective population size. Animal Conservation, 1(3), 173–177. 10.1111/j.1469-1795.1998.tb00026.x

[ece34155-bib-0009] Brown, W. D. , & Barry, K. L. (2016). Sexual cannibalism increases male material investment in offspring: Quantifying terminal reproductive effort in a praying mantis. Proceedings of the Royal Society B, 283(1833), 20160656 10.1098/rspb.2016.0656 27358366 PMC4936037

[ece34155-bib-0010] Buskirk, R. E. , Frohlich, C. , & Ross, K. G. (1984). The natural selection of sexual cannibalism. The American Naturalist, 123(5), 612–625. 10.1086/284227

[ece34155-bib-0011] Elgar, M. A. (1992). Sexual cannibalism in spiders and other invertebrates In Elgar M. A. & Crespi B. J. (Eds.), Cannibalism: Ecology and evolution among diverse taxa (pp. 128–155). Oxford: Oxford University Press.

[ece34155-bib-0012] Elgar, M. A. , Schneider, J. M. , & Herberstein, M. E. (2000). Female control of paternity in the sexually cannibalistic spider *Argiope keyserlingi* . Proceedings of the Royal Society of London B: Biological Sciences, 267(1460), 2439–2443. 10.1098/rspb.2000.1303 PMC1690835 11133035

[ece34155-bib-0013] Forster, L. M. (1992). The stereotyped behavior of sexual cannibalism in *Latrodectus hasselti* Thorell (Araneae, Theridiidae), the Australian redback spider. Australian Journal of Zoology, 40(1), 1–11. 10.1071/ZO9920001

[ece34155-bib-0014] Fromhage, L. , & Schneider, J. M. (2006). Emasculation to plug up females: The significance of pedipalp damage in *Nephila fenestrata* . Behavioral Ecology, 17(3), 353–357. 10.1093/beheco/arj037

[ece34155-bib-0015] Fromhage, L. , Uhl, G. , & Schneider, J. M. (2003). Fitness consequences of sexual cannibalism in female *Argiope bruennichi* . Behavioral Ecology and Sociobiology, 55(1), 60–64. 10.1007/s00265-003-0656-6

[ece34155-bib-0016] Gavín‐Centol, M. P. , Kralj‐Fišer, S. , De Mas, E. , Ruiz‐Lupión, D. , & Moya‐Laraño, J. (2017). Feeding regime, adult age and sexual size dimorphism as determinants of pre‐copulatory sexual cannibalism in virgin wolf spiders. Behavioral Ecology and Sociobiology, 71(1), 10 10.1007/s00265-016-2228-6

[ece34155-bib-0017] Grimm, V. (1999). Ten years of individual‐based modelling in ecology: What have we learned and what could we learn in the future? Ecological Modelling, 115(2), 129–148. 10.1016/S0304-3800(98)00188-4

[ece34155-bib-0018] Hassell, M. P. , Lawton, J. H. , & Beddington, J. R. (1976). The components of arthropod predation: I. The prey death‐rate. The Journal of Animal Ecology, 45(1), 135–164. 10.2307/3772

[ece34155-bib-0019] Hebets, E. A. (2003). Subadult experience influences adult mate choice in an arthropod: Exposed female wolf spiders prefer males of a familiar phenotype. Proceedings of the National Academy of Sciences, 100(23), 13390–13395. 10.1073/pnas.2333262100 PMC263824 14597702

[ece34155-bib-0020] Henriksson, S. (1997). Sexual cannibalism in the fishing spider and a model for the evolution of sexual cannibalism based on genetic constraints. Evolutionary Ecology, 11(3), 255–273.

[ece34155-bib-0021] Herberstein, M. E. , Gaskett, A. C. , Schneider, J. M. , Vella, N. G. F. , & Elgar, M. A. (2005). Limits to male copulation frequency: Sexual cannibalism and sterility in St Andrew's Cross spiders (Araneae, Araneidae). Ethology, 111(11), 1050–1061. 10.1111/j.1439-0310.2005.01114.x

[ece34155-bib-0022] Herberstein, M. , Schneider, J. , & Elgar, M. (2002). Costs of courtship and mating in a sexually cannibalistic orb‐web spider: Female mating strategies and their consequences for males. Behavioral Ecology and Sociobiology, 51(5), 440–446.

[ece34155-bib-0023] Hurd, L. E. , Eisenberg, R. M. , Fagan, W. F. , Tilmon, K. J. , Snyder, W. E. , Vandersall, K. S. , … Welch, J. D. (1994). Cannibalism reverses male‐biased sex ratio in adult mantids: Female strategy against food limitation? Oikos, 69, 193–198. 10.2307/3546137

[ece34155-bib-0024] Johnson, J. C. (2001). Sexual cannibalism in fishing spiders (*Dolomedes triton*): An evaluation of two explanations for female aggression towards potential mates. Animal Behaviour, 61(5), 905–914. 10.1006/anbe.2000.1679

[ece34155-bib-0025] Johnson, J. C. , & Sih, A. (2005). Precopulatory sexual cannibalism in fishing spiders (*Dolomedes triton*): A role for behavioral syndromes. Behavioral Ecology and Sociobiology, 58(4), 390–396. 10.1007/s00265-005-0943-5

[ece34155-bib-0026] Kralj‐Fišer, S. , Čandek, K. , Lokovšek, T. , Čelik, T. , Cheng, R. C. , Elgar, M. A. , & Kuntner, M. (2016). Mate choice and sexual size dimorphism, not personality, explain female aggression and sexual cannibalism in raft spiders. Animal Behaviour, 111, 49–55. 10.1016/j.anbehav.2015.10.013

[ece34155-bib-0027] Lawrence, S. E. (1992). Sexual cannibalism in the praying mantid, *Mantis religiosa*: A field study. Animal Behaviour, 43(4), 569–583. 10.1016/S0003-3472(05)81017-6

[ece34155-bib-0028] Lelito, J. P. , & Brown, W. D. (2006). Complicity or conflict over sexual cannibalism? Male risk taking in the praying mantis *Tenodera aridifolia sinensis* . The American Naturalist, 168(2), 263–269. 10.1086/505757 16874635

[ece34155-bib-0029] Maxwell, M. R. , Gallego, K. M. , & Barry, K. L. (2010). Effects of female feeding regime in a sexually cannibalistic mantid: Fecundity, cannibalism, and male response in *Stagmomantis limbata* (Mantodea). Ecological Entomology, 35(6), 775–787. 10.1111/j.1365-2311.2010.01239.x

[ece34155-bib-0125] Miller, K.A. , Nelson, N. J. , Smith, H.G. & Moore, J. A. (2009). How do reproductive skew and founder group size affect genetic diversity in reintroduced populations?. Molecular ecology, 18(18), 3792–3802. 19732338 10.1111/j.1365-294X.2009.04315.x

[ece34155-bib-0030] Newman, J. A. , & Elgar, M. A. (1991). Sexual cannibalism in orb‐weaving spiders: An economic model. The American Naturalist, 138(6), 1372–1395. 10.1086/285292

[ece34155-bib-0031] Persons, M. H. , & Uetz, G. W. (2005). Sexual cannibalism and mate choice decisions in wolf spiders: Influence of male size and secondary sexual characters. Animal Behaviour, 69(1), 83–94. 10.1016/j.anbehav.2003.12.030

[ece34155-bib-0032] Prenter, J. , MacNeil, C. , & Elwood, R. W. (2006). Sexual cannibalism and mate choice. Animal Behaviour, 71(3), 481–490. 10.1016/j.anbehav.2005.05.011

[ece34155-bib-0033] Pruitt, J. N. , Berning, A. W. , Cusack, B. , Shearer, T. A. , McGuirk, M. , Coleman, A. , … Singh, N. (2014). Precopulatory sexual cannibalism causes increase egg case production, hatching success, and female attractiveness to males. Ethology, 120(5), 453–462. 10.1111/eth.12216

[ece34155-bib-0034] R Core Team . (2017). R: A language and environment for statistical computing. Vienna, Austria: R Foundation for Statistical Computing Retrieved from https://www.R-project.org/.

[ece34155-bib-0035] Roggenbuck, H. , Pekár, S. , & Schneider, J. M. (2011). Sexual cannibalism in the European garden spider *Araneus diadematus*: The roles of female hunger and mate size dimorphism. Animal Behaviour, 81(4), 749–755. 10.1016/j.anbehav.2011.01.005

[ece34155-bib-0036] Schneider, J. M. , & Elgar, M. A. (2001). Sexual cannibalism and sperm competition in the golden orb‐web spider *Nephila plumipes* (Araneoidea): Female and male perspectives. Behavioral Ecology, 12(5), 547–552. 10.1093/beheco/12.5.547

[ece34155-bib-0037] Schneider, J. M. , & Lesmono, K. (2009). Courtship raises male fertilization success through post‐mating sexual selection in a spider. Proceedings of the Royal Society of London B: Biological Sciences, 276(1670), 3105–3111. 10.1098/rspb.2009.0694 PMC2817132 19515667

[ece34155-bib-0038] Schwartz, S. K. , Wagner, W. E. , & Hebets, E. A. (2016). Males can benefit from sexual cannibalism facilitated by self‐sacrifice. Current Biology, 26(20), 2794–2799. 10.1016/j.cub.2016.08.010 27720621

[ece34155-bib-0039] Spence, J. R. , Zimmermann, M. , & Wojcicki, J. P. (1996). Effects of food limitation and sexual cannibalism on reproductive output of the nursery web spider *Dolomedes triton* (Araneae: Pisauridae). Oikos, 75, 373–382. 10.2307/3545877

[ece34155-bib-0040] Walker, L. A. , & Holwell, G. I. (2015). Sexual cannibalism in a facultative parthenogen: The springbok mantis (*Miomantis caffra*). Behavioral Ecology, 27, 851–856.

[ece34155-bib-0041] Wilder, S. M. , & Rypstra, A. L. (2008). Sexual size dimorphism predicts the frequency of sexual cannibalism within and among species of spiders. The American Naturalist, 172(3), 431–440. 10.1086/589518 18616388

