# Supplementary figures and images for: Sexual cannibalism and population viability
Source: Ecol Evol. 2018 Jun 24;8(13):6663–70. doi: 10.1002/ece3.4155 (PMC6053559; doi:10.1002/ece3.4155)

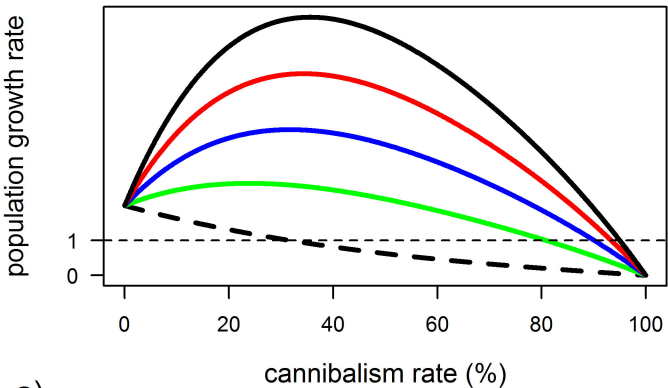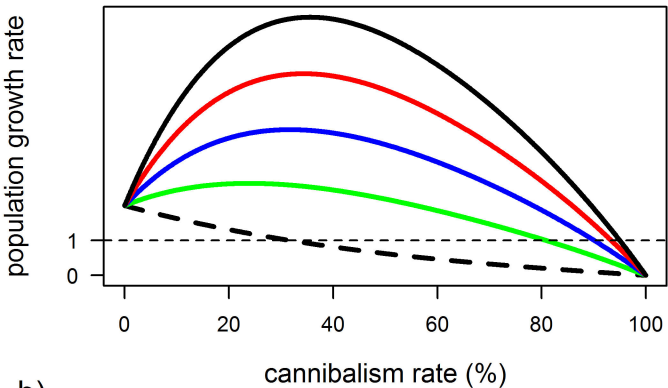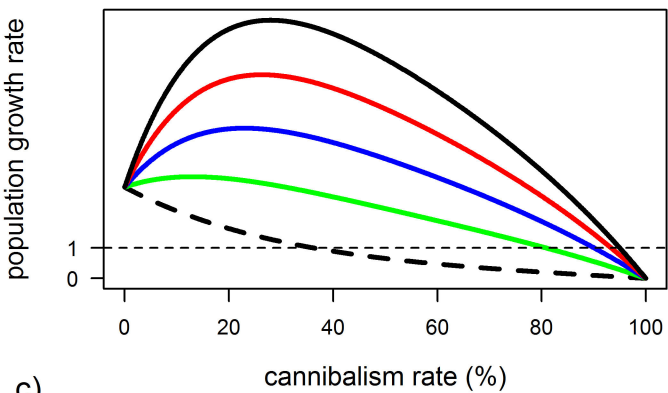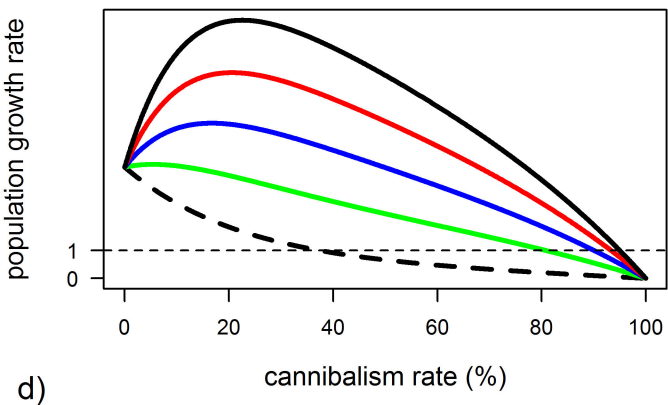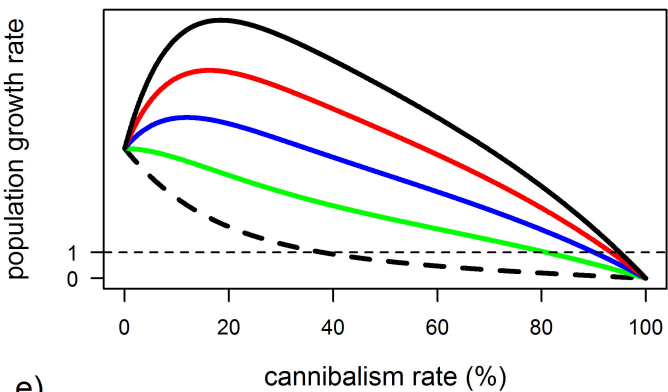

Supplement: Supplementary file 1 [file ECE3-8-6663-s001.pdf]

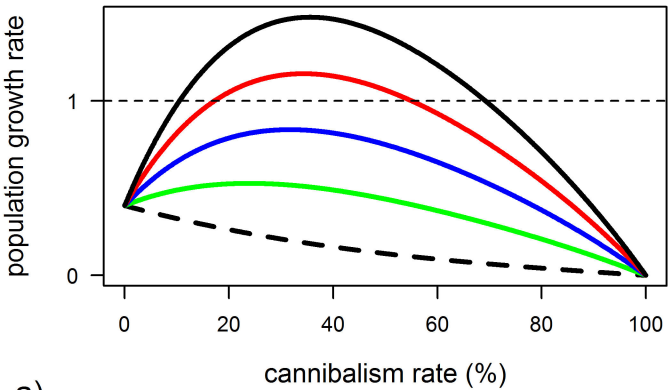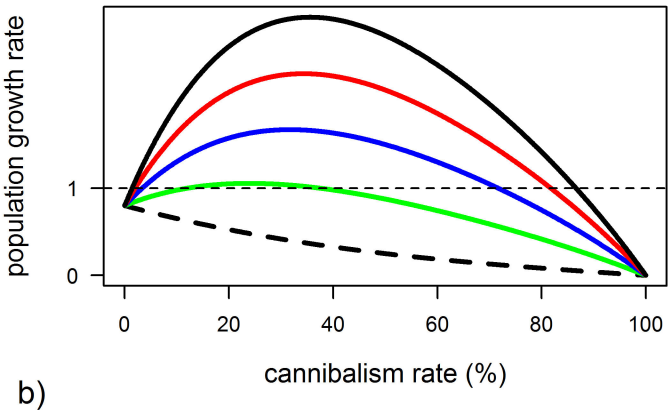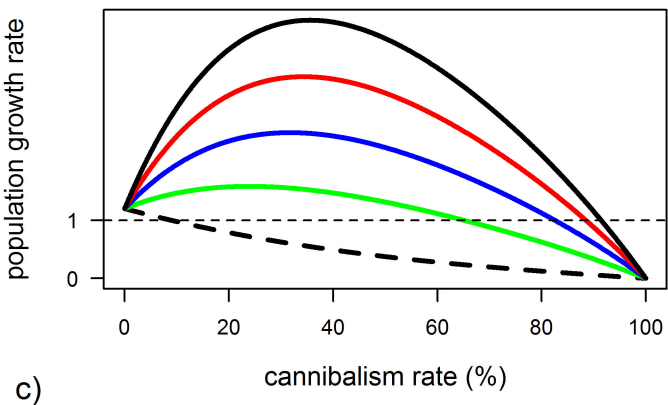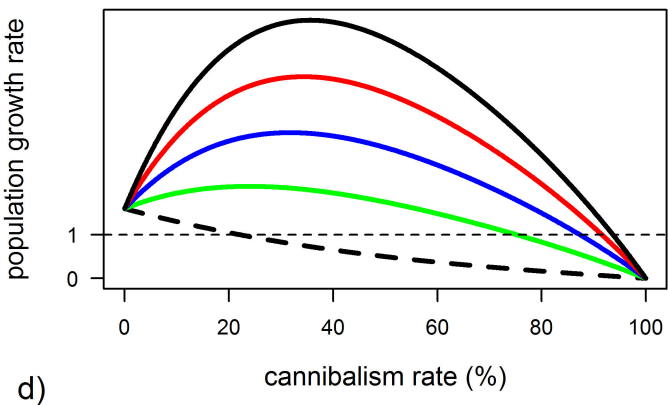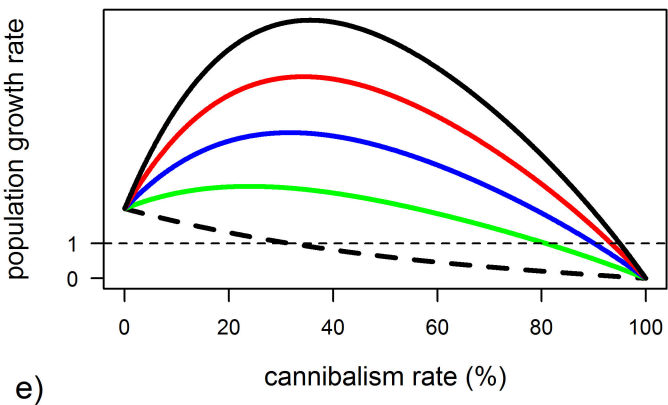

Supplement: Supplementary file 2 [file ECE3-8-6663-s002.pdf]
