# Supplementary material for: Sexual cannibalism and population viability
Source: Ecol Evol. 2018 Jun 24;8(13):6663–70. doi: 10.1002/ece3.4155 (PMC6053559; doi:10.1002/ece3.4155)
Supplement: Supplementary file 3 [file ECE3-8-6663-s003.docx]

**Supplementary material for Fisher et al. Sexual Cannibalism and Population Viability.**

*Varying maximum mating quota (h)*

The overall trends produced by our model were skewed to the left in response to increasing the value of *h* (supplementary fig. 1). As such, if the maximum mating rate for individuals is higher, the optimal rate of sexual cannibalism for population growth rate shifts to the left, towards lower rates of cannibalism. However, the overall trends produced by the model are not altered by changing the value of *h* (the curves are skewed but the relationship relative to cannibalism rate and parameter *b* remain the same).

**Supplementary figure 1** – The response of population growth rate to cannibalism rate and cannibalism-derived fecundity benefits (*b*) for five different values of the parameter defining the maximum number of matings per individual (*h*): a) *h* = 1, b) *h* = 2, c) *h* = 3, d) *h* = 4, and e) *h* = 5.

*Varying the probability that a male will get the opportunity to mate (k)*

Reducing parameter *k* below 1 creates a scenario in which male reproductive success is limited by something other than sexual cannibalism – intense male competition for example. Population growth rate was reduced in response to reducing the value of *k* (supplementary figure 2). However, the magnitude of this reduction was constant for all values of our other model parameters. As such, the relative trends produced by our model were not affected by changing the value of *k*.

**Supplementary figure 2** - The response of population growth rate to cannibalism rate and cannibalism-derived fecundity benefits (*b*) for five different values of the parameter defining the probability that a male could attempt to mate with a female (*k*) (*h*): a) *k* = 0.2, b) *k* = 0.4, c) *k* = 0.6, d) *k* = 0.8, and e) *k* = 1.
